# Relevant factors for early liver transplantation after Kasai portoenterostomy

**DOI:** 10.1186/s12887-020-02355-8

**Published:** 2020-10-20

**Authors:** Liang Ge, Jianghua Zhan, Wei Gao, Shengqiao Zhao, Xiaodan Xu, Ran Dou

**Affiliations:** 1 Graduate School of Tianjin Medical University, Tianjin, 300070 China; 2 Department of Pediatric Surgery, Tianjin Children’s Hospital, LongYan Road 238, Beichen District, Tianjin, 300134 PR China; 3 Department of Transplantation, Tianjin First Central Hospital, Tianjin, 300192 China

**Keywords:** Biliary atresia, Liver transplantation, Relevant factors, Kasai portoenterostomy

## Abstract

**Background:**

To explore the relevant factors for early liver transplantation (LT) after Kasai portoenterostomy (KP).

**Methods:**

Retrospective analysis was performed for 200 children with biliary atresia, who underwent LT with hepatic failure after KP. According to the interval between KP and LT, they were divided into three groups: G1 (≤6-month), G2 (6-month~ 2-year) and G3 (> 2-year). Gender, age of Kasai portoenterostomy, jaundice-clearance, cholangitis after KP and liver function indexes before LT were compared among the three groups.

**Results:**

The proportion of patients with age of KP (≤90-day) in G1 was lower than that in G3 (*P* = 0.003). Jaundice-clearance occurred in 6 (7.6%), 26(28.6%) and 26 (86.7%) patients after KP in G1, G2 and G3 respectively (*P* < 0.001). There were statistical differences in the incidence of early cholangitis, late cholangitis and repeated cholangitis among the three groups (*P* = 0.035, < 0.001 and 0.022). The native liver survival (NLS) rate of children at operation age > 90-day was lower than that of children at operation age ≤ 90-day (*P* = 0.002). The NLS rate of the children with jaundice-clearance after KP was significantly better than that of the children without jaundice-clearance (*P* < 0.001). The NLS rate of the children with early cholangitis after operation was lower than that in children without early cholangitis (*P* = 0.026). The NLS rate of patients of G2 and G3 with cholangitis after KP was lower than that in children without cholangitis (*P* = 0.017). Multiple logistic regression analysis showed uncleared jaundice after KP was a risk factor for the NLS time in patients.

**Conclusion:**

The age of KP (> 90-day), jaundice-unclear and early cholangitis could reduce the NLS time after KP, which were related to early liver transplantation. Jaundice-unclear was a risk factor for early liver transplantation.

## Background

Biliary atresia (BA), characterized by progressive inflammation and fibrous obstruction of hepatic bile ducts, is a serious hepatobiliary disease in infancy. It can lead to cholestasis, hepatic fibrosis and cirrhosis. Kasai portoenterostomy has been a primary operation for biliary atresia since professor Morio Kasai first performed in 1959. Shinkai et al. [1] reported that the 5-, 10-, and 20-year survival rates of patients with their native livers were 63, 54, and 44%, respectively. Nio [2] reported that 20-year native-liver survival rate was 49%. However, the native liver survival after KP in mainland China is not optimistic. Only less than 30% of the patients could achieve long-term survival with native liver after KP, and most of them eventually die or receive LT [3]. Although liver transplantation is constrained by the shortage of donor, high cost and lifelong use of anti-rejection drugs after operation, living donor liver transplantation for treating biliary atresia has made rapid progress in recent years. Among the patients who received LT after KP, some of them received LT within 6 months after KP [4], or even within 3 months [5]. Which factors lead to LT in a short time after KP? How to regulate these factors to improve the native liver survival time after KP? Is it necessary to avoid the attack of KP and directly perform LT for some patients who would have no effect after KP? The objective of this study is to identify the relevant factors for early LT after KP.

## Methods

A retrospective study was carried out for patients who underwent LT in Tianjin first central hospital from Dec 2011 to Mar 2019. Patients who received KP due to type III [6] biliary atresia were recruited. All of them were definitely diagnosed with end-stage liver disease, accompanying ascites, encephalopathy, gastrointestinal hemorrhage, repeated cholangitis or seriously affecting the quality of life. Liver pathology after LT confirmed liver cirrhosis, with pseudolobules, widened portal area and inflammatory cell infiltration. There were many bile emboli in the capillary bile duct.

A total of 8 variables from the clinical and laboratory database were obtained for study, including gender, age of KP, jaundice-clearance was defined as to achieve normal bilirubin level (less than 20 μmol/L) within 6 months post-Kasai [7], cholangitis (defined as fever, acholic stool, increase of clinical jaundice and bilirubin levels, or positive blood culture) [8], early cholangitis was defined as to occur within 1 month after KP, late cholangitis was defined as to occur 1 month after KP, repeated cholangitis defined as the occurrence of cholangitis more than three times in 6 months after KP and biochemical indicators of liver function (i.e. ALB, ALT, AST, DBIL, ALP and GGT) before liver transplantation were analyzed**.**

According to the native liver survival (NLS) time after KP (i.e. the interval between KP and LT), the patients were divided into three groups. The patients whose NLS time ≤ 6-month were included in G1, 6-month~ 2-year were included in G2, and > 2-year were included in G3. The NLS time ≤ 6-month was defined as early transplantation.

Statistical analysis was performed using standard statistical package (IBM Statistical Package for Social Science, version 20.0). Categorical data were analyzed using the chi-square test. Continuous data were compared using one-way ANOVA analysis or Kruskal-Wallis *H* test. Kaplan Meier survival analysis and Log-rank test were used for univariate analysis of NLS. Multiple logistic regression was used for multivariate analysis. A *P* value of < 0.05 was regarded as significant.

## Results

Two hundred patients who underwent open KP during Jul 2003 and Jul 2018 were enrolled. Eighteen of them performed in Tianjin Children’s Hospital, and the rest performed in other pediatric centers of mainland China. The results were summarized in Table 1.

**Table 1:** Demographics and overall results of patients recruited

Variables	*N* = 200 n (%)
Sex	
Male	93 (46.5)
Female	107 (53.5)
Age of KP (days)	
Mean (95%Cl)	64 (61–67)
Median (range)	62 (51–75)
Time of native liver survival (days)	
Mean (95%Cl)	436 (357–530)
Median (range)	212 (142–378)
Jaundice-clearance	58 (29)
Cholangitis	112 (56)
Early cholangitis	39 (19.5)
Late cholangitis	73 (36.5)
Repeated cholangitis	37 (18.5)

Among 200 BA patients, 79 of 200 (39.5%) in G1, 91 of 200 (45.5%) in G2 and 30 of 200 (15%) in G3. There were statistical differences in the proportion of age of KP (≤90-day) among the three groups(*P* = 0.011), and G1 was lower than G3(*P* = 0.003). Jaundice-clearance occurred in 6 (7.6%), 26(28.6%) and 26 (86.7%) patients after KP in G1, G2 and G3 respectively (*P* < 0.001), gradually elevated from G1 to G3. There were statistical differences in the incidence of early cholangitis among the three groups (*P* = 0.035). G3 was significantly lower than G1 (*P* = 0.009). There were statistical differences in the incidence of late cholangitis among the three groups (*P* < 0.001). G2 was significantly higher than G1 (*P* < 0.001). There were statistical differences in the incidence of repeated cholangitis among the three groups (*P* = 0.022). G3 was lower than the G2 (*P* = 0.023). The results were summarized in Table 2.

**Table 2:** Characteristics and clinical features of biliary atresia patients of three groups

Variables	G1 *N* = 79 n (%)	G2 *N* = 91 n (%)	G3 *N* = 30 n (%)	*P* value
Sex				
Male	40 (50.6)	36 (39.6)	17 (56.7)	0.169
Female	39 (49.4)	55 (60.4)	13 (43.3)	
Age of KP (days)				
Mean (95%Cl)	68 (63–72)	62 (58–67)	59 (53–64)	0.086
Median (range)	67 (55–83)	58 (51–74)	60 (47–72)	
≤ 90-day	66 (83.5)	80 (87.9)	30 (100)	**0.011**
> 90-day	13 (16.5)	11 (12.1)	0 (0)	
Jaundice-clearance	6 (7.6)	26 (28.6)	26 (86.7)	**<0.001**
Cholangitis	37 (46.8)	64 (70.3)	11 (36.7)	**0.001**
Early cholangitis	20 (25.3)	18 (19.8)	1 (3.3)	**0.035**
Late cholangitis	17 (21.5)	46 (50.5)	10 (33.3)	**<0.001**
Repeated cholangitis	11 (13.9)	24 (26.4)	2 (6.7)	**0.022**

There were significant differences in ALT among the three groups (*P* = 0.006), which was higher in G1 than in G3 (*P* = 0.007). There were significant differences in AST, ALP and GGT among the three groups (*P* < 0.001, < 0.001, 0.001), which were higher in G1 and G2 than in G3 (*P* < 0.05). There were significant differences in DBIL among the three groups (*P* < 0.001), with G1 to G3 decreasing gradually (*P* < 0.05). The results were summarized in Table 3.

**Table 3:** Biochemical indicators before liver transplantation of three groups

Variables	G1 *N* = 79	G2 *N* = 91	G3 *N* = 30	*P* value
ALB(g/L)				0.194
Mean (95%Cl)	34.15 (33.04–35.24)	35.54 (34.48–36.69)	35.56 (33.33–37.72)	
Median (range)	34 (31–37)	35 (35–39)	36.5 (31.83–39.33)	
ALT(U/L)				**0.006**
Mean (95%Cl)	153.21 (125.86–187.17)	144.39 (110.62–193.26)	94.26 (70.35–121.35)	
Median (range)	122 (75–190)	94 (53–176)	59.9 (45.05–130.13)	
AST(U/L)				**<0.001**
Mean (95%Cl)	259.03 (221.93–302.61)	243.73 (202.25–291.52)	148.39 (104–201.13)	
Median (range)	220 (143–331)	172 (120–334)	102.5 (65.25–177.25)	
DBIL (μmol/L)				**<0.001**
Mean (95%Cl)	205.46 (179.69–230.96)	161.48 (129.01–191.06)	78.16 (39.71–121.75)	
Median (range)	204 (118–253)	132 (28–257)	24.39 (12.61–68.25)	
ALP(U/L)				**<0.001**
Mean (95%Cl)	733.78 (650.55–823.34)	713.25 (632.08–791.79)	443.16 (370.15–521.84)	
Median (range)	644 (449–944)	605 (427–858)	421.35 (282.5–596.28)	
GGT(U/L)				**0.001**
Mean (95%Cl)	469.24 (390.49–547.96)	377.37 (313.79–445.49)	228.22 (154.05–316.72)	
Median (range)	375 (174–634.5)	287 (146–551)	162 (93.53–281)	

**Fig. 1:**
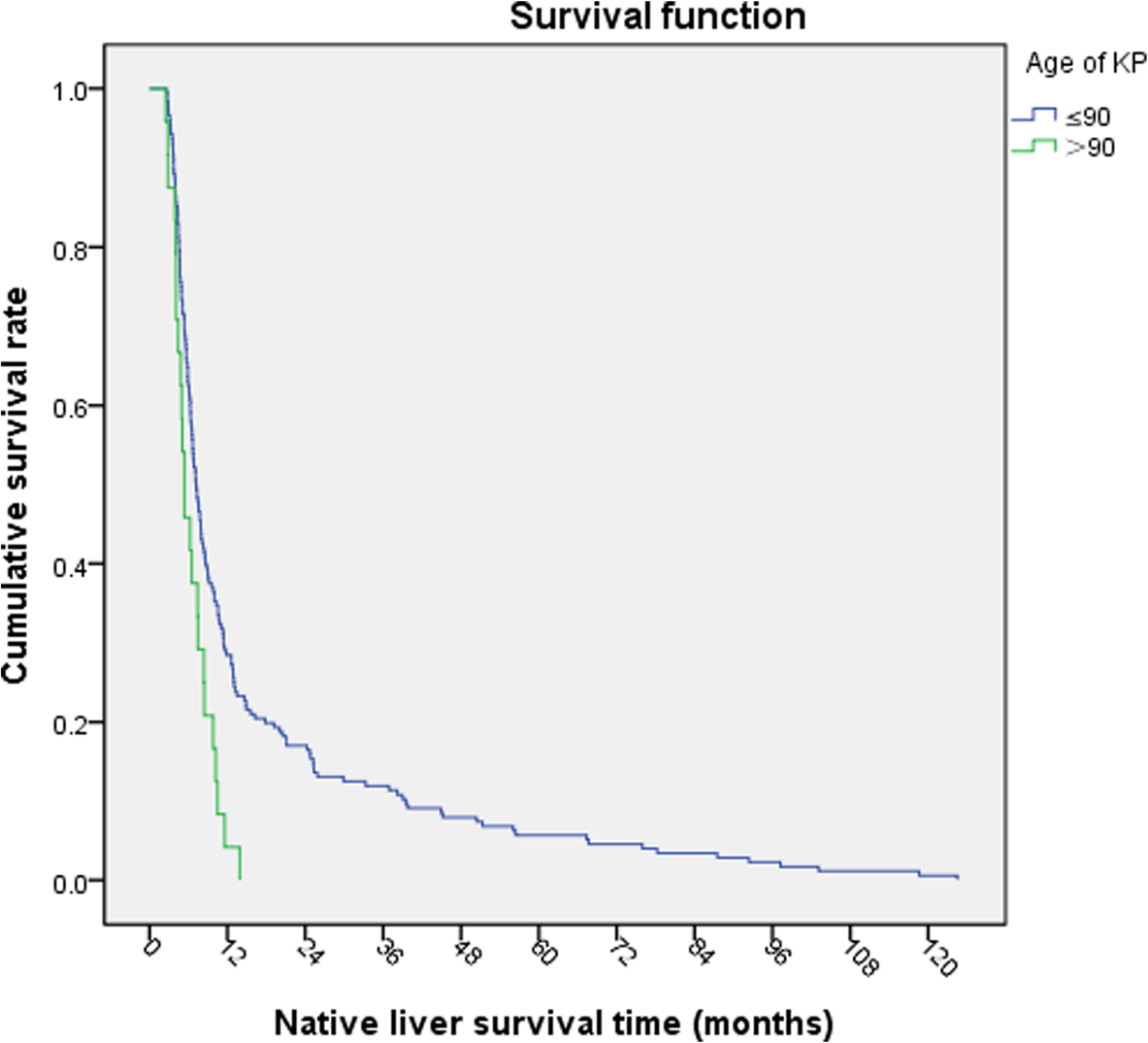
NLS rate at different age of KP

**Fig. 2:**
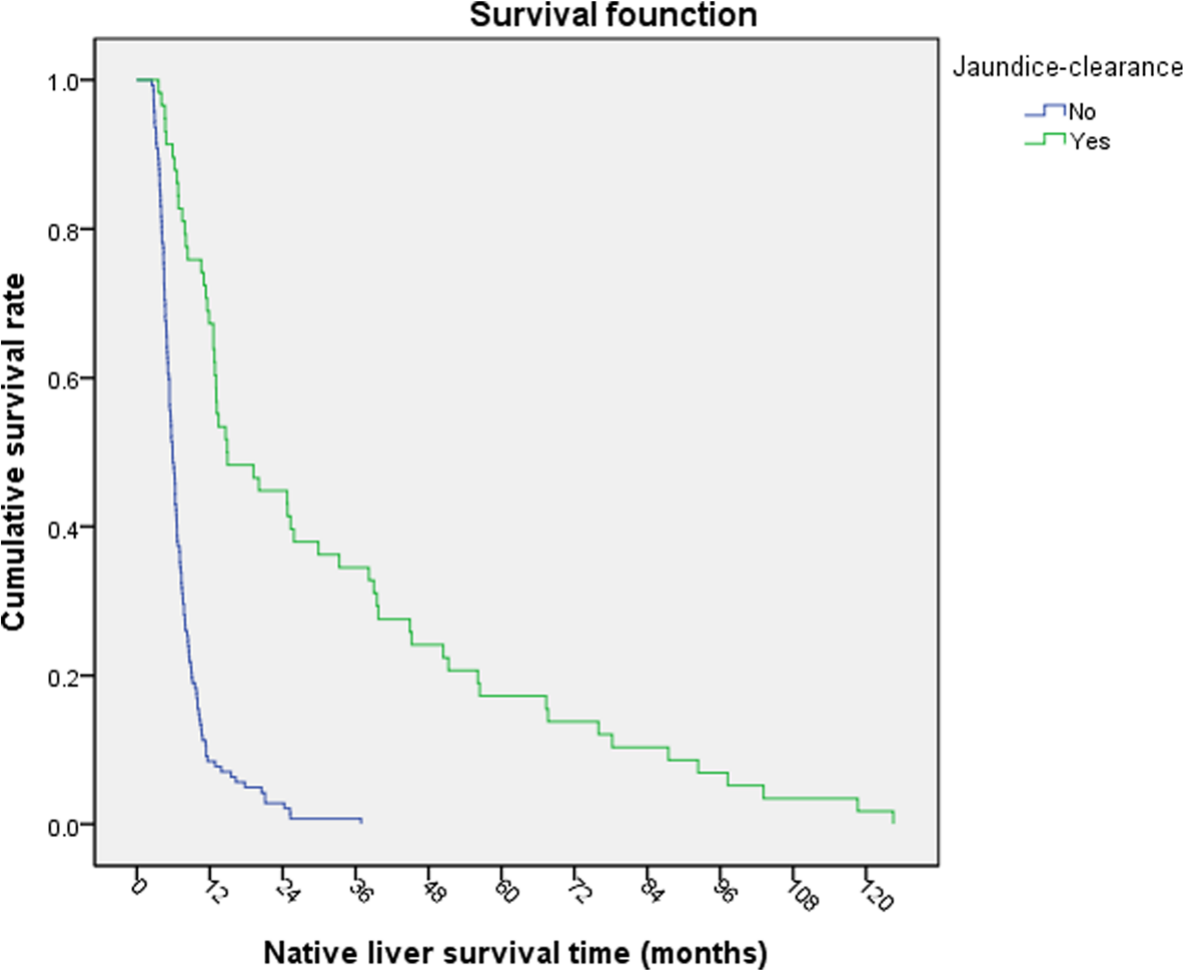
NLS rate with or without jaundice clearance

**Fig. 3:**
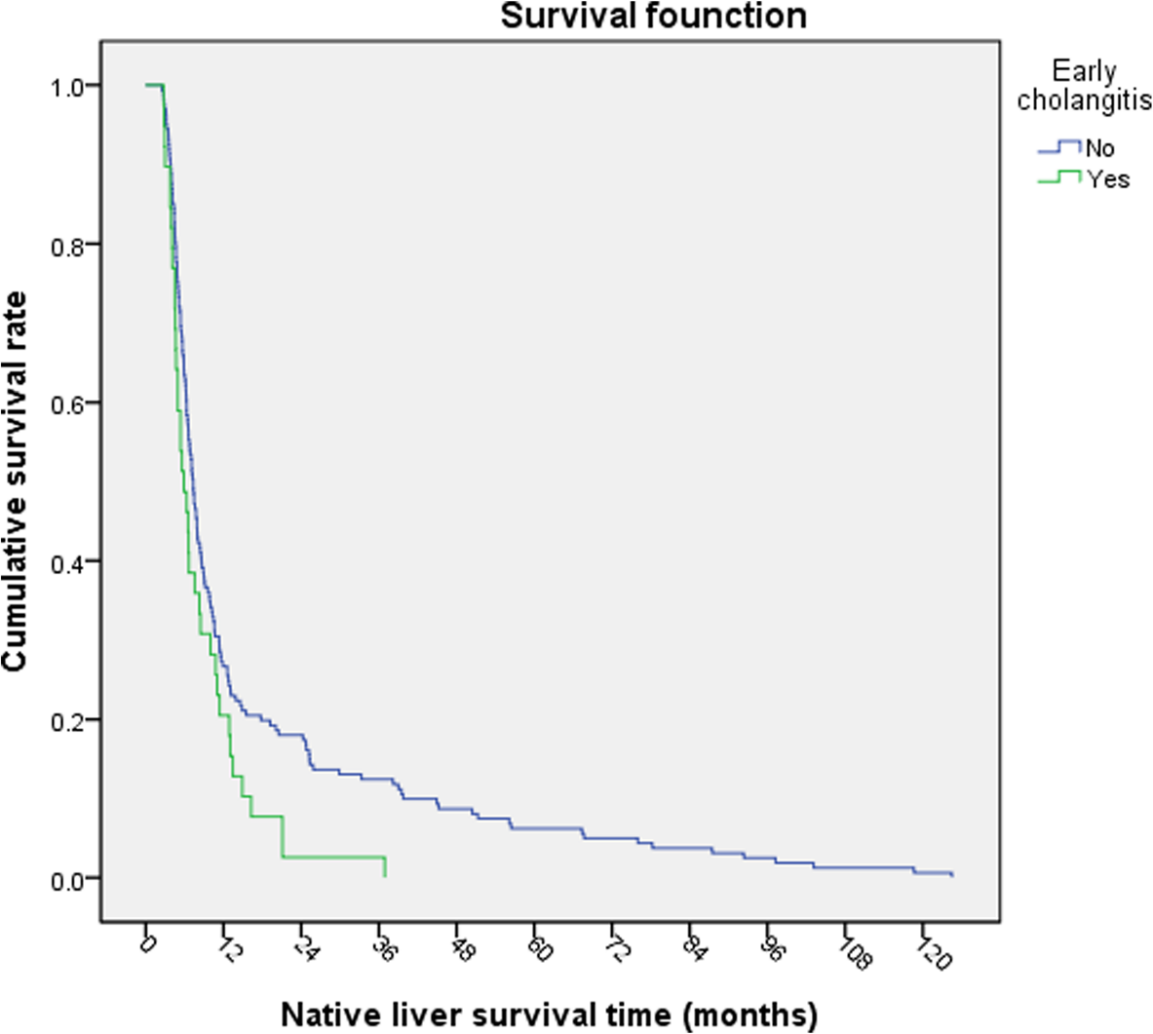
NLS rate with or without early cholangitis

**Fig. 4:**
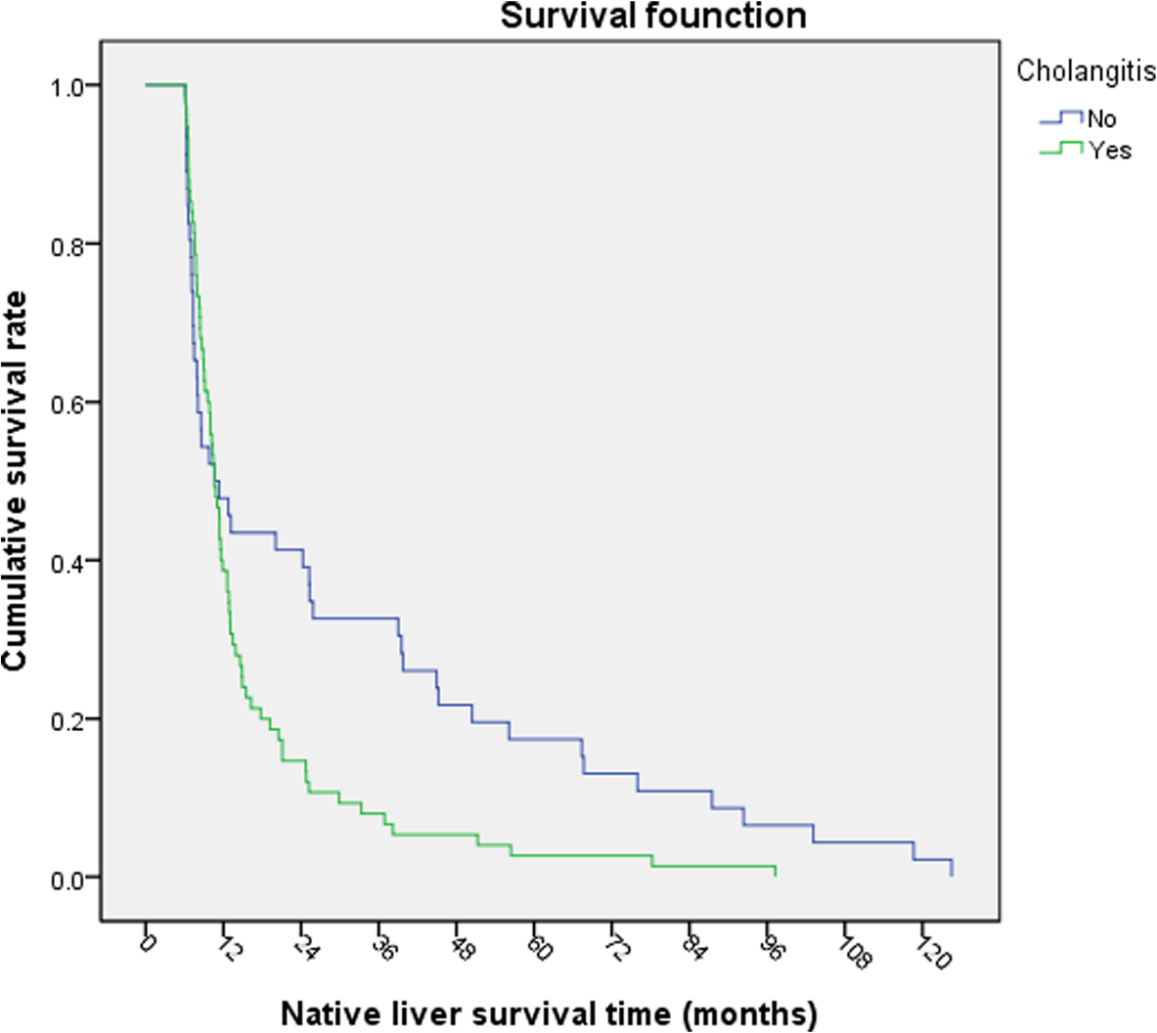
NLS rate with or without cholangitis(G2 and G3)

Kaplan Meier survival analysis was carried out. The NLS rate of children at operation age > 90-day was lower than that of children at operation age ≤ 90-day (Log-rank*χ*^2^ = 9.221, *P* = 0.002, Fig. 1). The NLS rate of the children with jaundice-clearance after KP was significantly better than that of the children without jaundice-clearance (Log-rank *χ*^2^ = 75.541, *P* < 0.001, Fig. 2). The NLS rate of the children with early cholangitis after operation was lower than that in children without early cholangitis (Log-rank *χ*^2^ = 4.931, *P* = 0.026, Fig. 3). There was no significant difference in native survival rate between those with cholangitis and without cholangitis (Log-rank*χ*^2^ = 0.0214, *P* = 0.644). However, the NLS rate of patients of G2 and G3 with cholangitis after KP was lower than that in children without cholangitis (Log-rank *χ*^2^ = 5.736, *P* = 0.017, Fig. 4). There was no significant difference in the NLS rate of patients between with or without late cholangitis after KP (Log-rank *χ*^2^ = 1.26, *P* = 0.262). So is the repeated cholangitis (Log-rank *χ*^2^ = 0.082, *P* = 0.775).

Multiple logistic regression analysis was conducted. The age of KP (≤90-day and > 90-day), the jaundice-clearance and the early cholangitis after KP were taken as independent variables. The NLS time (G1, G2 and G3) was taken as dependent variable. The results showed that the uncleared jaundice after KP was a risk factor for the NLS time in children, which could shorten the NLS time and promote early LT. The results were summarized in Table 4.

**Table 4:** Multivariate analysis for early LT in children after KP

Variables		β	SE	Wald	*P*	Exp(β)	Exp(β) 95%Cl	
							down	up
G2	Intercept	0.898	0.680	1.743	0.187	–	–	–
	Early cholangitis	0.312	0.383	0.663	0.415	1.367	0.645	2.897
	Jaundice-clearance	−1.575	.485	10.522	**0.001**	0.207	0.080	0.536
	Age of KP(≤90-day)	0.373	0.460	0.657	0.418	1.452	0.589	3.581
G3	Intercept	−18.430	1.148	257.663	0.000	–	–	–
	Early cholangitis	2.169	1.130	3.688	0.055	8.752	0.956	80.099
	Jaundice-clearance	−4.784	0.864	30.643	**0.000**	0.008	0.002	0.045
	Age of KP(≤90-day)	18.128	0.000	–	–	74597740.461	74597740.461	74597740.461

The reference category was G1

## Discussion

In this report, the biochemical indicators of liver function (ALT, AST, TBIL, DBIL, ALP and GGT) of children undergoing early LT were higher than those with advanced LT. Pathological examination confirmed liver cirrhosis in all patients. Therefore, it is suggested that the liver function deteriorates rapidly after KP in patients undergoing early LT. Current studies have found that factors affecting KP’s prognosis were age of operation, jaundice-clearance, cholangitis and liver pathological changes [2, 3, 5, 9–11]. There is a close relationship between KP prognosis and liver transplantation. Therefore, this study explores the risk factors of early LT after KP by analyzing the factors that may affect the prognosis of KP.

Biliary atresia is a progressive disease. The earlier diagnosed, the lighter hepatic fibrosis and infiltration of inflammatory cells around the bile ducts, the prognosis after surgical treatment is much better theoretically. The 20-year survival rate of native liver in patients with age at initial Kasai operation less than 70 days was significantly higher than those more than 70 days (50.88% vs 28.57%) [1]. Nio et al. [10] classified 242 patients with type III biliary atresia into six groups based on their age at KP, and demonstrated that the age at portoenterostomy and the jaundice clearance rate were significantly inversely correlated. Schreiber et al. [12] found that the 10-year native liver survival rate of patients undergoing the Kasai operation at age ≤ 30 days was 49%, while only 15% in > 90 days group. It was found that in patients with age ≤ 90 days at the Kasai operation, the 1-year, 3-year, and 5-year NLS rates were 92.3, 69.5, and 54.3% versus 68.7, 49.8, and 25.4% in patients with age > 90 days, respectively [9]. However, Chen et al. [8] found that the 2-year survival rate of native liver had no difference in operation age > 90 days group comparing to others. Some patients with biliary atresia older than 90 days of age are still capable of achieving long-term survival with their native livers after undergoing the Kasai procedure. This study showed that the NLS rate in patients with age of KP (> 90-day) was lower than those age of KP(≤90-day). Therefore, there is a correlation between the age of KP and the NLS after KP. The age of KP should not be later than 90 days.

Jaundice-clearance after Kasai operation indicates the decrease of serum total bilirubin level after KP, which can be used to evaluate the biliary drainage after KP and as a sign of success of KP. The shorter time (≤60 days) to achieve jaundice-free after KP, the more benefit to reduce liver damage and slow down the development of liver fibrosis, so as to obtain a better native liver survival [11]. Post-KP BA patients who became jaundice free within less than 60 days of KP seemed to be capable of NLS in the long term with the possibility of survival without LT [13]. This study showed that the jaundice-clearance rate of patients with advanced LT was significantly higher than that of patients with early LT. Survival analysis showed that the survival rate of native liver in patients with jaundice-clearance was significantly better than that in patients without jaundice-clearance. Multivariate analysis showed that the jaundice-clearance after KP was a risk factor for the survival of native liver after KP. More than 90% of the children who underwent LT within 6 months after KP did not achieve effective bile drainage, which led to liver failure and cirrhosis rapidly. The reason may be that some patients have poor reaction to KP and can’t establish effective biliary drainage after operation. This is consistent with the poor effect of KP on biliary atresia with congenital malformations (especially splenic malformations) and cytomegalovirus infection reported in the literature [14]. In addition, this study can suggest that liver have progressed to severe fibrosis when performing KP in G1. So, we don’t recommend to perform KP if liver biopsy showed severe fibrosis because of poor prognosis. The patients were performed Kasai procedure in different hospitals, some of which lacked surgical experience. Non-standardized Kasai surgery would lead to deterioration of the disease. So centralized management is necessary.

Cholangitis is the most common and serious complication after KP. The time of occurrence and frequency of cholangitis are closely related to prognosis. Studies have shown that the earlier cholangitis occurs after KP, the worse the prognosis is [3, 11]. Early cholangitis after KP resulted in rapid occlusion of bile ducts due to inflammation and scar, obstruction of bile drainage, and aggravation of liver damage. Regarding the disease onset, there was a higher percentage of intractable cholangitis occurring within the first year of Kasai operation, destroying bile drainage and leading to end-stage liver failure [15]. In this study, the incidence of early cholangitis decreased gradually from G1 to G3. Survival analysis showed that the survival rate of native liver in patients with early cholangitis was lower than that in patients without early cholangitis. Therefore, early cholangitis after KP is correlated with early LT, which is in line with the above literatures.

The occurrence of post-operative cholangitis is associated with the NLS after surgery. Patients with postoperative cholangitis was associated with a decreasing of 1-year, 3-year, and 5-year NLS rates from 91.7, 75.8 and 75.8% to 80.1, 50.7 and 23.3%, respectively [9]. The only significant prognostic factor associated with poor short and long-term outcomes is the presence of repeated cholangitis [16]. The more frequently and severely cholangitis occurs after KP, the worse bile drainage, the more severe liver fibrosis and the shorter native liver survival time [17, 18]. Adversely, the rates of cholangitis, late cholangitis and repeated cholangitis in G2 were higher than those in G1. It may be related to the age of liver transplantation were too young in G1, or to insufficient sample size. However, survival analysis in children who underwent LT 6 months after KP (G2 and G3) showed that cholangitis after KP could reduce the survival time of native liver and promote liver transplantation.

## Conclusions

The age of KP (> 90-day), jaundice-unclear and early cholangitis could reduce the NLS time after Kasai portoenterostomy, which were related to early liver transplantation. Jaundice-unclear was a risk factor for early liver transplantation. Therefore, we should improve the early diagnosis rate and operation rate of children with biliary atresia, standardize KP and management, improve the clearance rate of jaundice and reduce the incidence of cholangitis, so we can improve the NLS rate and postpone the time for LT. However, for some children with biliary atresia, such as those with embryonic type, malformation, cytomegalovirus infection and severe fibrosis, even early Kasai procedure can’t reconstruct enough bile drainage, it may be much more benefit to avoid the impact of Kasai operation and directly perform liver transplantation instead. This requires further research and a more valuable evaluation system.

## Data Availability

The datasets used and/or analysed during the current study are available from the corresponding author on reasonable request.

## Abbreviations

LT: Liver transplantation
KP: Kasai portoenterostomy
NLS: Native liver survival
BA: Biliary atresia
ALB: Albumin
ALT: Alanine aminotransferase
AST: Aspartate aminotransferase
DBIL: Direct bilirubin
ALP: Alkaline phosphatase
GGT: Glutamyltranspeptidase
